# A Case of Dislocation and Fracture of the Head of the Femur

**Published:** 1871-02

**Authors:** L. A. Harcourt

**Affiliations:** Chicago, Ill.


BUFFALO

Medical and Surgical Journal.

VOL. X. FEBRUARY, 1871. No. 7.

### Original Communications.

ART. I.—*A Case of Dislocation and Fracture of the Head of the Femur.* By L. A. HARCOURT, M. D., Chicago, Ill.

On Saturday evening, Nov. 26th, 1870, Mr. F. W., Seigel St., aged 41 years, whilst driving a one horse wagon, collided with an express, and was thrown to the pavement, striking upon the outer and slightly anterior aspect of the left trochanter major, the wheel passing over him. He was taken to his home, where I saw him an hour after the accident occurred.

His left foot was everted, the corresponding limb shortened about an inch, the left groin swollen and tender, and the outer aspect of the hip somewhat flattened. The limb was fixed, admitting of neither external or internal rotation, neither could it be flexed. There was no crepitus. The groin was so exquisitely painful, that the patient would not tolerate the manipulations which I attempted to make, with a view of determining the presence or absence of the head of the femur in that position. Here, the deformity of direction was such as might obtain either in a fracture of the cervix femoris, or in a forward dislocation on the pubis; while the deformity of contour favored the dislocation, and the immobility of the limb seemed to contra-indicate the existence of fracture. It is true, the accident would be more likely to produce fracture than dislocation; and yet the force being applied to the outer and anterior aspect of the trochanter, would have a tendency to force it backwards and inwards, and thus cause the head of the femur to infringe against the inner and upper margin of the acetabulum; and, if the force were sufficient, to override it, thereby producing this form of dislocation. Such, at least, was my impression.

Having administered an anæsthetic, I seized the limb, (an assistant steadying the pelvis,) and flexed it upon the pelvis, then abducting it to relax the outer portion of the capsular ligament, and drawing or rather lifting it outwards and downwards, a change took place in the relation of the parts, which I conceived to be a reduction of the dislocation. There was just such a sound as we would expect from the head of the bone passing into the acetabulum—just such a sound as I have often heard when dislocations were reduced. Every one in the room heard it, and the patient himself had consciousness enough to know that he was relieved. He instantly exclaims: Doctor, I am all right now! I then extended the limb, and the deformity disappeared; nor did it manifest any disposition to return. I simply placed a bandage around the thighs. Before flexing the limb, I should have felt for the head of the bone; for, if found on the pubis below Poupart's ligament, there could be no doubt about the diagnosis.

The next day, (Sunday,) I called to see him, and looked carefully for shortening, still suspecting the existence of fracture. None could be discovered. The patient was comfortable—the thigh somewhat swollen. Monday noon, called again. There was apparent shortening of an inch, the foot slightly everted. Measurement of both limbs proved that it was real. I then told him the neck of the femur was fractured, and as he could not pay for treatment, sent him to the County Hospital, under charge of Prof. Powell of Rush Medical College. The Doctor confirmed my diagnosis as to fracture; but, from the symptoms *then* existing, could not say that there had been a dislocation. I am satisfied, however, that there was a dislocation, partial or complete. The fracture was either impacted or only partial—that is, the fragments were not separated; hence its characteristic signs were absent, and its existence obscure. Had my manipulations on the limb simply disengaged the impacted fragments, the shortening and deformity would have returned as soon as they were discontinued. But they did not return, hence the fragments were not disengaged by the manipulations, but by the subsequent movements of the patient. When the shortening did recur, I could easily reduce it by simply making extension on the foot; but it returned the instant the extension ceased.

The treatment consisted in simply placing the man on his back, and making extension by means of weights applied in the usual way. Small pillows or pads were placed at the sides of the limb to prevent displacement by rotation. The result was better than could be hoped for. In five weeks union had taken place. In six weeks the patient could sit up in bed; and now, eight weeks from the occurrence of the accident, he can walk about the room on crutches, and sustain the weight of the body on the limb. The limb is shortened 3-8 of an inch, the trochanter slightly tilted forward; but there is no visible deformity. For all practical purposes it will be as good as ever it was. Dr. Powell does not pretend to say whether the fracture was within or without the capsular ligament, neither do I; but, whether within or without, the result was all that could be desired.

The case, on the whole, is somewhat anomalous, yet serves to show that, in a patient of good constitution, the accident is not as grave as some authors would have us believe.

